# Evaluation of effectiveness of 10% polyvinylpyrrolidone-iodine solution against infections in wire and pin holes for Ilizarov external fixators

**DOI:** 10.1590/S1516-31802005000200005

**Published:** 2005-03-02

**Authors:** Adelina Morais Camilo, José Carlos Bongiovanni

**Keywords:** Infection, Ilizarov technique, Povidone iodine, Bone wires, Sodium chloride, Infecção, Técnica de Ilizarov, Povidona-iodo, Fios ortopédicos, Cloreto de Sódio

## Abstract

**CONTEXT AND OBJECTIVE:**

Superficial infection at wire and pin insertions in the skin is a frequent disorder among patients utilizing the Ilizarov method. The objective of this study was to evaluate the effectiveness of daily topical application of 10% polyvinylpyrrolidone-iodine solution against infections of the holes for Kirschner wires and Schanz pins among patients using Ilizarov external fixators, in comparison with cleaning these holes only with 0.9% sterile physiological saline solution.

**DESIGN AND SETTING:**

Controlled randomized clinical trial, in the Orthopedics and Traumatology Outpatient Clinic, Hospital São Paulo, and Orthopedics and Traumatology Center of Jundiaí.

**METHODS:**

30 patients were treated using the Ilizarov technique: 15 were instructed to apply 0.9% physiological saline dressing on the wire and pin insertions and 15 to apply 0.9% physiological saline plus 10% polyvinylpyrrolidone-iodine. Patients were evaluated at outpatient return visits for identification of signs and symptoms of superficial infection at wire and pin insertion sites. Samples were collected from cases of purulent exudate secretion, for culturing and clinical tests.

**RESULTS:**

The chi-squared and Fischer exact tests were applied, but no statistically significant association between the intervention of topical polyvinylpyrrolidone-iodine solution and the prevention of infections at wire and pin insertions could be found.

**CONCLUSIONS:**

Topical 10% polyvinylpyrrolidone-iodine solution applied daily to Kirschner wire and Schanz pin insertions did not reduce the incidence of superficial infection at these holes, in comparison with mechanical removal of dirt using 0.9% physiological saline solution.

## RESUMO

**CONTEXTO E OBJETIVO:**

A incidência de infecção superficial no trajeto dos fios e pinos junto à pele é uma complicação freqüente em pacientes em tratamento pelo método de Ilizarov. Este estudo objetivou avaliar a eficácia da aplicação diária da solução tópica de polivinilpirrolidona-iodo a 10% na interferência de infecção nos orifícios dos fios de Kirschner e pinos de Schanz de pacientes em uso de fixador externo de Ilizarov comparada à limpeza desses orifícios somente com soro fisiológico a 0,9%.

**TIPO DE ESTUDO E LOCAL:**

Ensaio clínico randomizado controlado, no Ambulatório de Ortopedia e Traumatologia do Hospital São Paulo e Centro de Ortopedia e Traumatologia de Jundiaí.

**MÉTODOS:**

30 pacientes foram tratados pelo método de Ilizarov: 15 orientados a realizarem o curativo dos orifícios dos fios e pinos com soro fisiológico a 0,9% e 15 com soro fisiológico a 0,9% mais polivinilpirrolidona-iodo tópico a 10%. Os pacientes foram avaliados nos retornos ambulatoriais, para identificação dos sinais e sintomas de infecção superficial nos orifícios dos fios e pinos. Amostras foram obtidas para cultura e antibiograma na presença de exsudato com pus à expressão.

**RESULTADOS:**

Aplicado os testes exato de Fisher e qui-quadrado não foi possível encontrar associação estatisticamente significante da intervenção da solução tópica de polivinilpirrolidona-iodo na prevenção de infecções nos trajetos dos fios e pinos.

**CONCLUSÕES:**

A solução de polivinilpirrolidona-iodo tópica a 10% quando aplicada diariamente no orifício dos fios de Kirschner e pinos de Schanz à pele não reduz a incidência de infecção superficial nesses trajetos quando comparada com pacientes que realizam a remoção mecânica de sujidades com soro fisiológico a 0,9%.

## INTRODUCTION

Several studies have shown that skin infections adjacent to wire and pin insertions are frequent complications of the Ilizarov method. Such infections have been associated with loss of wire tension, abscess formation around the wire or pin, presence of necrotic tissue all along the wire or pin and bad cleaning of the pins and wires when applying dressings.^1–3^

The presence of foreign bodies in the organism irritates tissues and favors infection. Foreign bodies cause severe inflammatory reactions.^4^ In cases of infection, good performance by the multidisciplinary staff and the patient is needed to promote the recovery process and prevent complications.

The use of topical antimicrobial drugs to avoid skin infections adjacent to the wire and pin insertions of the fixator is not very effective because the infection is often polymicrobial. The antimicrobial drugs of choice must be wide-spectrum, hypoallergenic and present reduced side effects.^5^

Today, a topical antiseptic solution of 10% polyvinylpyrrolidone-iodine is, according to the Brazilian Ministry of Health,^6^ the only acceptable formulation for use in dressings. Free iodine is the active agent in polyvinylpyrrolidone-iodine, and it has a wide antibacterial spectrum, acting on Gram-positive and Gram-negative bacteria, fungi, yeast, viruses and protozoa. Polyvinylpyrrolidone-iodine is a non-irritating, painless and non-corrosive water-soluble product, and does not alter thyroid function tests. It forms a pellicle when applied to the skin, penetrating hair follicles and starting to act four minutes after application.^7^

The purpose of the present study was to evaluate the effectiveness of topical polyvinylpyrrolidone-iodine solution in comparison with the use of only 0.9% saline, with regard to mechanical and healing cleaning of the skin adjacent to the insertions of Kirschner wires and Schanz pins in the prophylaxis of superficial infection at wire and pin insertions.

## MATERIAL AND METHODS

This study was designed as a clinical controlled randomized trial. The participants were 30 patients who were treated using the Ilizarov external fixation technique and were followed up as outpatients. They were registered at two different orthopedics services: 13 patients at the Department of Orthopedics and Trauma-tology of Universidade Federal de São Paulo, Escola Paulista de Medicina, a tertiary-level private institution, and 17 at the Orthopedics and Traumatology Center of Jundiaí (OTC), a private institution. The follow-up took place between March 2001 and September 2002. The team of surgeons of the external fixators group of Universidade Federal de São Paulo acts in both of these services, which made it possible to keep the same procedures for all the patients before, during and after the surgery. Thirty-one patients were initially contacted, of whom 30 joined the project and concluded an adequate number of steps to generate results for use in the study.

This study had prior approval from the Research Ethics Committee of Universidade Federal de São Paulo and all the participants freely signed an informed consent statement.

The patients selected were having their first postoperative return visit after the insertion of an Ilizarov external fixator, with follow-up by the external fixators group of Universidade Federal de São Paulo, Escola Paulista de Medicina, or by the Orthopedics and Traumatology Center of Jundiaí. Patients presenting active infection in the limb that had received the external fixator were excluded from this study.

### Randomization

The patients eligible for this study were randomly assigned to one of two groups (A or B) with different dressing application instructions, by means of the use of 30 consecutively numbered sealed envelopes, 15 for each group. At the time of the first outpatient return visit following application of the Ilizarov external fixator, the patients received the envelope, which was opened according to the sequential numbers identified, and the patients were given the guidance indicated in it.

Group A (control) – patients were told to apply the dressing after taking a shower and washing their hands with clean water and soap. After drying their hands with a clean towel they were to start applying the dressing. At each of the pin and wire insertion sites of the Ilizarov external fixator, the skin was to be cleaned with sterile gauze soaked in 0.9% physiological saline solution. After removing all dirt, the insertion sites were to be dried using gauze and individually covered with folded gauze. After being given oral instructions, the patients received a folder containing illustrations and written instructions.

Group B – patients were told to apply the dressing after taking a shower and washing their hands with clean water and soap. After drying their hands with a clean towel they were to start applying the dressing. At each of the pin and wire insertion sites of the Ilizarov external fixator, the skin was to be cleaned with sterile gauze soaked in 0.9% physiological saline solution. After removing all dirt, the insertion sites were to be treated using gauze soaked with polyvinylpyrrolidone-iodine (PVPI). After PVPI application, all excess was to be removed using dry gauze and each insertion site was to be covered with folded gauze. After being given oral instructions, the patients received a folder containing illustrations and written instructions.

The study was conducted by analyzing the incidence of skin infection at the insertion sites for the fixator wires and pins, in the two groups.

### Follow-up

During the outpatient return visits, the nurse assessed the presence or absence of signs and symptoms of an infectious process at the pin and wire sites. Material was also collected for the culturing of secretions, when drainage of purulent exudate was observed at the wire or pin sites of the Ilizarov external fixator.

When material was collected for culturing, the wire and pin sites were irrigated with 0.9% sterile physiological saline until all visible fragments had been removed. All exfoliated, loose or necrotic material was removed with the aid of sterile gloves and gauze. Sterile gauze was then applied to remove all excess sterile physiological saline.

While using sterile gloves, the nurse exposed the margins of the wire or pin insertions from which the material would be collected, using the thumb and index finger of the left hand. This allowed the insertion of a swab around the wire and pin. After collection, the set of swabs was identified by name, hospital registration, date and time of collection and anatomical region. The material was immediately forwarded to Hospital São Paulo for culturing and testing in the laboratory. None of the patients were using antibiotics at the time when the samples were collected.

The presence of purulent exudate was the criterion adopted for defining the presence of an infectious process.

### Statistical analysis

The chi-squared or nonparametric Fisher exact test was used to compare two categorized variables in the statistical analysis. The Mann-Whitney and Student t tests were also used. The Access and Excel computer software were used. In all tests, results with a risk of less than 0.05 or 5% were considered significant.

From the 30 patients included in the study, 15 (50%) were randomly chosen to apply dressings to wire and pin holes with sterile physiological saline solution and the other 15 to apply dressings with 0.9% physiological saline solution plus topical 10% polyvinylpyrrolidone-iodine. The patients were followed up throughout the time they were using the Ilizarov external fixator, which ranged from 95 to 726 days, with a mean of 273.

## RESULTS

All patients were Caucasians. Males were predominant in the polyvinylpyrrolidone-iodine solution dressing group (73.3). In the control group there was a predominance of females (53.3%).

The mean age was 31 years (range: 14-59) in the polyvinylpyrrolidone-iodine dressing group and 32 years (range: 14-53) in the control group. No statistically significant difference regarding social characteristics was observed between the groups of the study population (Table 1).

**Table 1:** Social characteristics of 30 patients using Ilizarov external fixators who had dressings with or without PVP-I

Characteristics	Physiological saline + PVP-I (n =15)	%	Control (n = 15)	%	p*
**Sex (n)**					
Male	11	73.3	7	46.7	
Female	4	26.7	8	53.3	0.136
**Age (years)**					
Mean	31		32		
Range	14-59		14-53		0.742
**Race (n)**					
Caucasian	15	100	15	100	

*PVP-I = polyvinylpyrrolidone-iodine.*

There was no significant difference between the sites that were being treated using the Ilizarov method in either study group. The sites most used in the polyvinylpyrrolidone-iodine group were the femur and tibia in three cases (20%) and tibia in eight (53.3%), while in the control group, the most common were the tibia in nine cases (60%) and femur and tibia in four (26.7%). Regarding the reasons for the treatment among the 15 patients who had dressings using polyvinylpyrrolidone-iodine, five cases (33.3%) were for bone lengthening, three (20%) for lengthening plus compression, three (20%) for strengthening plus transport, one (6.67%) for compression, two (13.33%) for transport plus compression and one (6.67%) for transport associated with compression and lengthening. Among the patients in the control group, two (13.33%) used the treatment for bone lengthening, two (13.33%) for lengthening plus tension, six (40%) for lengthening plus transport, two (13.33%) for compression, one (6.67%) for bone transport, one (6.67%) for transport plus compression and one (6.67%) for transport associated with compression and lengthening. No significant difference was observed between the patients in the polyvinylpyrrolidone-iodine dressing group and the controls.

The presence of fever, hyperemia, edema, pain and purulent exudate was evaluated for all the patients during the visits. Of the 30 patients evaluated, three (10%) presented hyperthermia, 17 (56.7%) hyperemia, 17 (56.67%) edema, 30 (100%) pain and 17 (56.67%) purulent exudate at the wire and pin sites of the Ilizarov external fixator (Table 2). No significant difference between the groups in relation to signs and symptoms of infection at the wire and pin sites was observed.

**Table 2:** Distribution of patients who presented some type of infection at the wire and pin insertion (fever, hyperemia, edema, pain and purulent exudate) of Ilizarov external fixators at visit to nurse

Group				
Infection signs and symptoms (absent/present)		PVP-I	Control	P
Fever	Absent	13 (86.7%)	14 (93.3%)	1.000*
	Present	2 (13.3%)	1 (6.7%)	
Hyperemia	Absent	0 (0.0%)	0 (0.0%)	
	Present	15 (100%)	15 (100%)	
Edema	Absent	5 (33.3%)	8 (53.3%)	0.269**
	Present	10 (66.7%)	7 (46.7%)	
Purulent secretion	Absent	5 (33.3%)	8 (53.3%)	0.269**
	Present	10 (66.7%)	7 (46.7%)	
Pain	Absent	14 (93.3%)	13 (86.7%)	1.000*
	Present	1 (6.7%)	2 (13.3%)	

* *= Descriptive level of probability for Fisher's exact test;*

** *= Descriptive level of probability for the chi-squared test;*

*PVP-I = polyvinylpyrrolidone-iodine.*

Seven purulent exudate cultures were collected from six patients presenting exudate, from the skin adjacent to the wire and pins of the Ilizarov external fixator. Four cultures were collected from patients in the group that applied 0.9% physiological saline plus topical polyvinylpyrrolidone-iodine dressings and three (75%) were collected from the skin at the wire sites and one (25%) was collected from the skin at the pin sites. Of the three cultures collected from patients in the group that applied dressings with physiological saline, only one (33.33%) was collected from the skin at the wire sites and two (66.67%) from the skin at the pin sites.

Among the 17 patients who evolved with superficial infection at the wire and pin insertions, five (29.41%) were rehospitalized, of whom four received endovenous antibiotic therapy and in one case the Kirschner wire was removed due to infection (Table 3). No significant difference between the groups was observed regarding infection and rehospitalization.

**Table 3:** Distribution of 30 patients with Ilizarov external fixators, in relation to rehospitalization due to development of superficial infection at the wire and pin insertion

Group				
	Absent/present	PVP-I	Physiological Saline	p*
Infection	Absent	5 (33.3%)	8 (53.3%)	0.269
	Present	10 (66.7%)	7 (46.7%)	
Rehospitalization	Absent	11 (73.3%)	14 (93.3%)	0.330
	Present	4 (26.7%)	1 (6.7%)	

(*) *Descriptive level of probability for Fisher's exact test;*

*PVP-I = polyvinylpyrrolidone-iodine.*

In a subjective analysis, we asked the patients whether they were very satisfied or unsatisfied in relation to their visit to the nurse during all outpatient returns. In this survey, 29 (96.67%) were very satisfied, one (3.33%) was satisfied and none was unsatisfied.

## DISCUSSION

In spite of the great developments in medicine, infections are considered to be one of the most serious disorders that health professionals and patients have to deal with every day. They become especially dramatic in orthopedic surgery, because of the use of implants, which make the tissue vulnerable to the infectious process.

Several surgical techniques have been proposed in the literature for decreasing the rate of infection at the wire and pin sites of Ilizarov external fixators.^8^ However, there is still a scarcity of studies on nursing interventions that could be brought to bear for resolving such complications.

With regard to epidemiological aspects, our study showed that males were more affected than females. This was not, however, significant because more men underwent such surgery than women. A higher infection rate among younger patients was found, which is not in accordance with other studies. Graça et al.^10^ studied orthopedic surgery performed in the Pedro Ernesto university hospital and observed that there were greater numbers of infections among older patients.

According to Guarniero,^3^ the increase in the infection rate is directly related to the presence of soft tissue between the skin and the bone, the wire diameter and the instability of bone fixation. In our study, we found instability in two cases, of which one evolved with superficial infection and the other with reabsorption. Among the 17 patients who evolved with superficial infection at the wire and pin insertion, 53% developed infection in the region of the limb with the external fixator where there was greater presence of soft tissue (proximal region of the limb).

Thirteen patients who developed infection at the wire and pin site underwent bone lengthening at some time during their treatment. Aldegheri^9^ and Paley^11^ confirmed that excessive mobility between wire and pin is related to the appearance of superficial infection at the wire and pin insertion.

Guarniero^3^ reported that the greater the diameter was, the greater the probability of developing superficial infection at the wire and pin insertion was. In our study, among the 17 patients with superficial infection at the wire and pin insertion sites, seven developed infection at Schanz pin insertions, eight at Kirschner wire insertions and two at both wire and pin insertions. No statistically significant difference was found between the calibers of the materials used.

Collection of material for culturing of purulent exudate secretions was difficult, because of the small caliber of the hole into which the wires and pins are inserted. Swabs could not pass through these holes. Among the 17 patients presenting superficial infection at the wire and pin insertions, seven samples were collected from patients who, after the mechanical cleaning of the hole, continued to present drainage of exudate. Out of our cases, only one of the seven samples was considered inadequate for laboratory analysis. Giordano et al.^5^ reported that the sample contamination factor when collecting exudate from the wire and pin sites for culturing could be considered to be a critical factor in the final evaluation of a study.

Oestreicher and Tschantz^12^ compared the effect of 10% polyvinylpyrrolidone-iodine solution and 0.9% NaCl solution on the irrigation of contaminated wounds and did not find any significant differences between their two groups. Ribeiro et al.^7^ compared the use of 10% polyvinylpyrrolidone-iodine solution and 0.9% NaCl in dressings for surgical incisions and were unable to observe bacterial growth in either of their two groups. This led them to conclude that care in handling dressings and strictness regarding the surgical technique are effective means for the prophylaxis of infection. No significant difference was observed between the group who applied the polyvinylpyrrolidone-iodine dressing and the controls with only 0.9% physiological saline dressing in the present study.

When infection at wire and pin sites was identified in our study, the nurse was instructed to increase the frequency of dressing changes at the infection site. The medical team started clinical treatment using antibiotics as soon as possible. All patients evolved with remission of the inflammatory process, thereby avoiding complications such as deep infection and osteomyelitis.

At the end-of-treatment assessment, 29 patients (96.67%) reported that the nursing care during postoperative return visits following insertion of the Ilizarov external fixator helped to promote maintenance of self-care regarding the healing of the limb during the treatment. Oren^13^ reported that the role of the nurse is to encourage the patient to become a self-care agent. Campedelli and Friedlanter^14^ reported that the visit to the nurse is an activity that empowers her to act directly and independently towards the patient, thus emphasizing her professional autonomy.

## CONCLUSION

The results obtained allowed us to conclude that, when topical polyvinylpyrrolidone-iodine solution was applied to the Kirschner wire and Schanz pin insertions every day, among patients using an Ilizarov external fixator, this procedure did not reduce the incidence of superficial infection, in comparison with patients performing cleaning of the insertion using only 0.9% physiological saline solution.

Studies aimed at identifying a topical antimicrobial drug of wide bacteriological spectrum that could act to prevent infection at wire and pin sites need to be developed.
